# Secretomes from Mesenchymal Stem Cells against Acute Kidney Injury: Possible Heterogeneity

**DOI:** 10.1155/2018/8693137

**Published:** 2018-12-16

**Authors:** Kenji Tsuji, Shinji Kitamura, Jun Wada

**Affiliations:** Department of Nephrology, Rheumatology, Endocrinology and Metabolism, Okayama University Graduate School of Medicine, Dentistry, and Pharmaceutical Sciences, 2-5-1 Shikata-cho, Okayama 700-8558, Japan

## Abstract

A kidney has the ability to regenerate itself after a variety of renal injuries. Mesenchymal stem cells (MSCs) have been shown to ameliorate tissue damages during renal injuries and diseases. The regenerations induced by MSCs are primarily mediated by the paracrine release of soluble factors and extracellular vesicles, including exosomes and microvesicles. Extracellular vesicles contain proteins, microRNAs, and mRNAs that are transferred into recipient cells to induce several repair signaling pathways. Over the past few decades, many studies identified trophic factors from MSCs, which attenuate renal injury in a variety of animal acute kidney injury models, including renal ischemia-reperfusion injury and drug-induced renal injury, using microarray and proteomic analysis. Nevertheless, these studies have revealed the heterogeneity of trophic factors from MSCs that depend on the cell origins and different stimuli including hypoxia, inflammatory stimuli, and aging. In this review article, we summarize the secretomes and regenerative mechanisms induced by MSCs and highlight the possible heterogeneity of trophic factors from different types of MSC and different circumstances for renal regeneration.

## 1. Introduction

Acute kidney injury (AKI) is a worldwide healthcare problem associated with higher risks of mortality and increased length of hospitalization as well as the risk of chronic kidney disease and end-stage renal failure [1, 2]. In spite of the recent medical advances, few interventions are available other than supportive modalities, such as renal replacement therapies, against AKI. On the other hand, kidney has the ability to regenerate itself after AKI and some patients recover renal function after AKI [3]. Many researchers have tried to elucidate the mechanisms of renal regeneration. Over the past few decades, mesenchymal stem cell- (MSC-) based therapy represents the remarkable strategy to reconstitute the renal tubular formations and attenuate renal function after AKI.

MSCs are multipotent cells with the ability to differentiate into mesodermal lineages, including bone, muscle, chondrocyte, and adipocyte [4]. MSCs can be established from different tissues, such as bone marrow, adipose tissue, synovial tissue, umbilical cord, and connective tissue [4]. MSCs have shown to ameliorate tissue damages caused by renal injuries and diseases. Initially, researchers focused on the differentiation potential of MSCs against renal injury. Actually, MSCs were able to replace renal tubular cells and acquire an epithelial phenotype after renal injury in a murine renal injury model [5]. Nevertheless, the focus on the regenerative effects of MSCs has shifted into their ability to secrete trophic factors. MSCs secrete varieties of cytokines, chemokines, and growth factors as well as extracellular vesicles (EVs) that contain microRNAs (miRNAs), mRNAs, and proteins. Recent reports suggest that the therapeutic activity of MSCs is mainly mediated by the paracrine effect of secretomes. In the past few decades, many studies have identified these secretomes from MSCs and revealed the therapeutic mechanisms associated with cell proliferation, autophagy, cell apoptosis, tissue fibrosis, and inflammation. Meanwhile, recent reports imply the heterogeneity of secretomes of MSCs isolated from different origins. In addition, some reports have revealed that different kinds of stimuli affect the secretomes from MSCs. These differences might result in the different outcomes induced by the treatment with MSCs.

In this review article, we summarize the current knowledge about secretomes from MSCs and the therapeutic effects on renal injury and discuss about the possible heterogeneity caused by the differences of cell origins and stimuli.

## 2. MSC-Derived Soluble Protein for Renal Generation

MSCs have been reported to secrete a number of soluble factors including cytokines, chemokines, and growth factors for tissue regeneration. A number of groups have examined proteomic analysis of MSC secretomes to identify regenerative factors against tissue injury. These factors include angiogenic factors [6–8], chemokines [8–10], cytokines [6, 8, 10–13], growth factors [8, 10–12], and other proteins [10, 14–17] (Table 1). In general, these proteins exert many biological functions including cell growth, migration, inflammation, apoptosis, and fibrosis. In fact, under the status of kidney injuries, these factors contribute to renal regeneration through antiapoptosis, anti-inflammation, antifibrosis, matrix remodeling, and increased tubular cell proliferation. In addition, a number of reports demonstrate the paracrine effect of MSCs against renal injury. For example, Rota et al. revealed that human amniotic fluid-derived MSCs attenuate cisplatin-induced renal injury through the secretion of trophic factors, such as IL-6, VEGF, and IGF-1 [18]. Lv et al. demonstrated that MSCs ameliorate diabetic glomerular fibrosis via the secretion of BMP-7 [19]. Taken together, MSCs contribute to renal regeneration through the paracrine effect of soluble proteins from MSCs.

## 3. MSC-Derived Extracellular Vesicles: Exosomes and Microvesicles

Recent studies demonstrated that the secreted membrane vesicles called EVs play essential roles on intercellular communications [20–22]. EVs contain various molecules including proteins, miRNAs, and mRNAs. EVs can be organized into several categories such as exosomes (30-100 nm diameter), microvesicles (100-1000 nm diameter), and apoptotic bodies (50-5000 nm diameter) [23]. Increasing evidences have suggested that the MSC-derived EVs might be one of the major contributors for renal regeneration after AKI. Recent analyses identified proteins, miRNAs, and mRNAs in the EVs from MSCs by proteomic and microarray analysis.

## 4. mRNAs in MSC-Derived Extracellular Vesicles for Renal Regeneration

mRNAs from EVs transfer into target cells and act via translation into proteins as well as via competing RNAs to regulate stability, localization, and translational activity of mRNAs in target cells [24, 25]. Bruno et al., for the first time, demonstrated the therapeutic effect of MSC-derived EVs in glycerol-induced AKI in severe combined immunodeficiency (SCID) mice [26]. Using reverse transcription- (RT-) PCR arrays, they showed that human bone marrow MSC-derived EVs contain mRNAs involved in transcription (e.g., *CLOCK*, *IRF6*, and *LHX6*), immune regulation (e.g., *CRLF1*, *IL1RN*, and *MT1X*), cell cycle regulation (e.g., *SENP2*, *RBL1*, and *CDC14B*), DNA/RNA binding (e.g., *HMGN4*, *TOPORS*, and *ESF1*), actin cytoskeleton regulation (e.g., *DDN*, *MSN*, and *CTNNA1*), and extracellular matrix remodeling (e.g., *COL4A2*, *IBSP*) as well as cell differentiation into neuron (e.g., *RAX2*, *OR11H12*), bone (e.g., *NIN*, *BMP15*), endothelium/epithelium (e.g., *MAGED2*, *CEACAM5*), and hematopoietin (e.g., *HK3*, *EPX*). Importantly, the effect of EVs on the recovery from AKI was similar to the effect of MSCs, suggesting that the therapeutic effect by MSCs is mainly mediated by the MSC-derived EVs. In addition, pretreatment of RNase into MSC-derived EVs abolished the therapeutic effects by MSC-derived EVs, implying that RNAs from MSCs might be the main contributor for renal regeneration. Eirin et al. also characterized the mRNA cargo of EVs from porcine adipose tissue-derived MSCs by high-throughput RNA sequencing [27]. They revealed that EVs from MSCs contain mRNAs involved in transcription (e.g., *MDFIC*, *POU3F1*), angiogenesis (e.g., *HES1*, *TCF4*), adipogenesis (e.g., *CEBPA*, *KLF7*), and transforming growth factor- (TGF-) *β* signaling (e.g., *TGFB1*, *TGFB3*). In comparison with these two studies of mRNA analysis (Bruno et al.: 43 genes; Eirin et al.: 182 genes), only 1 overlap of mRNA was observed [28], suggesting the possible heterogeneity of mRNAs of EVs from different types of MSCs.

## 5. MicroRNAs in MSC-Derived Extracellular Vesicles for Renal Regeneration

miRNAs are one of the non-protein-coding RNAs that regulate gene expressions. In mammals, miRNAs are predicted to control approximately 30% of all protein-coding genes and have shown to contribute to majority of cellular processes [29]. MSC-derived EVs contain a number of miRNAs. Ferguson et al. indicated the biological processes and pathways modulated by miRNAs from MSC-derived EVs using the NanoString profiling of miRNAs from EVs [30]. They revealed that enriched miRNAs regulate target gene transcriptions associated with Wnt signaling, profibrotic signaling via TGF-*β* and PDGF, cell proliferation, and antiapoptosis. The top 23 miRNAs account for 79.1% of total miRNAs present in MSC-derived exosomes, and the remaining 148 miRNAs were at a very low ratio, suggesting that the top 23 miRNAs have predominant effects. These 23 miRNAs, miR-1246, miR-23a-3p, miR-451a, miR-125b-5p, miR-199a-3p/199b-3p, let-7a-5p, miR-4454/7975, miR-21-5p, let-7b-5p, miR-100-5p, miR-29a-3p, miR-144-3p, miR-29b-3p, miR-22-3p, miR-630, miR-221-3p, let-7i-5p, miR-424-5p, miR-191-5p, miR-25-3p, miR-130a-3p, miR-376a-3p, and miR-27b-3p, were predicted to target 5481 genes using the microRNA Data Integration Portal (miRDIP). Among these miRNAs, miR-29, let-7, miR-451, miR-630, miR-191, miR-21, and miR-22 are overlapped in other reports on miRNA analysis from MSC-derived EVs [27, 31–38] (Table 2).

Although the role of miRNAs has just recently begun to be analyzed, emerging evidences indicate that MSC-derived miRNAs have essential roles on tissue regeneration. For example, the let-7 family has been shown to repress multiple genes involved in cell cycle, cell apoptosis, and cell proliferation, including *CCNA2*, *CDC34*, *AURA/STK6*, *AURKB/STK12*, *E2F5*, and *CDK8* [39]. In addition, the Let-7 family has been shown to switch macrophages to the M2-like profile by targeting the toll-like receptor (TLR) 4 [33] as well as the induction of osteogenic differentiation [36]. Furthermore, Wang et al. reported that the overexpression of let-7c from MSCs attenuates kidney injury and downregulates fibrotic markers, such as collagen IV*α*1, TGF-*β*1, and TGF*β*R1, in a unilateral ureteral obstruction (UUO) model [40]. miR-125 has been reported to promote endothelial cell angiogenesis [41] while miR-29 has been reported to inhibit MCL-1 expression, an antiapoptotic protein [42], as well as ZFP36, which is an anti-inflammatory gene [43]. In fact, miR-29b inhibits the apoptotic pathway in doxorubicin-induced cardiotoxicity [44]. Furthermore, miR-29b attenuates angiotensin II-induced epithelial-mesenchymal transition (EMT) of rat renal tubular epithelial cells through the PI3K/AKT signaling pathway [45]. miR-21 is one of the miRNAs identified for the first time in mammals. miR-21 silences PTEN and GSK3b and reduces NF*κ*B activity, which induces inflammation [46]. In addition, miR-21 ameliorates ischemia/reperfusion- (I/R-) induced AKI by preventing epithelial cell apoptosis and inhibiting the maturation of dendritic cells [47]. Taken together, miRNAs from MSCs might be one of the major contributors for promoting renal regeneration.

## 6. Proteins in MSC-Derived Extracellular Vesicles for Renal Regeneration

In addition to the soluble factors from MSCs, MSC-derived EVs contain proteins that directly transfer into recipient cells. Kim et al. analyzed the EV-contained proteins from human bone marrow MSCs and identified 730 proteins [48]. Using functional analysis by the Database for Annotation, Visualization and Integrated Discovery (DAVID) software, they indicated that these proteins are involved in cell proliferation, cell adhesion, cell migration, and the regulation of cell morphogenesis. They also highlighted the trophic proteins, including surface receptors (e.g., PDGFRB, EGFR, and PLAUR), signaling molecules (e.g., MAPK1, CDC42, RRAS/NRAS, and VAV2), and cell adhesion (e.g., EZR, FN1, IQGAP1, CD47, integrins, and LGALS1/LGALS3) and MSC-associated proteins (e.g., CD9, CD63, CD81, CD109, CD151, CD248, and CD276). Another group also analyzed the proteins in EVs from human embryonic and bone marrow-derived MSCs, revealing that EVs contain trophic proteins associated with angiogenesis (VEGF, angiopoietin), inflammation (TNF-inducible gene 6 protein (TNFAIP6)), and TGF-*β* signaling [49–51]. Taken together, MSC-derived EVs contain a number of trophic proteins that have the potential for the promotion of tissue regeneration.

## 7. MSC-Derived EV Therapy in Experimental AKI Models

Emerging evidences have shown that treatment with MSC-derived EVs attenuates renal injury after AKI in a variety of murine models (Table 3). For example, Bruno et al. revealed that human bone marrow MSC-derived EV injection improves renal function and tubular injury in a glycerol-induced AKI rat model through the stimulation of tubular cell proliferation and inhibition of cell apoptosis [26, 52]. With the fact that the pretreatment with RNase into MSC-EVs reversed the therapeutic effects, these trophic mechanisms might be induced by RNAs in MSC-EVs. They also examined using another AKI model of cisplatin-induced AKI, indicating similar therapeutic effects including decreased renal cell apoptosis and preserved renal function [53].

Gatti et al. applied human bone marrow MSC-derived EVs into an I/R-induced AKI model, revealing that MSC-EV treatment attenuates I/R-induced AKI by reducing cell apoptosis and increasing renal tubular cell proliferation [54]. These trophic effects were abolished by RNase treatment similar to the study by Bruno et al. [26], reinforcing the concept that the trophic effect by MSC-EV treatment is mainly mediated by the mRNAs and/or miRNAs in MSC-EVs. Furthermore, they demonstrated that MSC-EV treatment also inhibits the progression of subsequent chronic kidney disease after AKI.

Zhou et al. showed that the injection of human umbilical cord MSC-derived EVs into the renal capsule attenuates cisplatin-induced AKI by improving oxidative stress as well as the inhibition of tubular cell apoptosis and necrosis [55]. Likewise, Zhang et al. reported that MSC-EV treatment protects against I/R-induced AKI through antioxidation possibly by enhancing NF-E2-related factor 2 (Nrf2)/antioxidant responsive element (ARE) activation [56]. They also reported that MSC-EV treatment ameliorates oxidative stress and renal injury in I/R-induced AKI through the decreased expression of NOX2 and reactive oxygen species (ROS) [57]. Furthermore, decreased renal fibrosis was observed with MSC-EV treatment. Zou et al. also reported decreased renal fibrosis with the treatment of human umbilical cord MSC-derived EVs as well as the downregulation of CXCL1 and decrease of CD68^+^ macrophage [58]. They also reported the increased expression of renal VEGF with the treatment of MSC-EVs as well as decreased renal fibrosis, indicating that MSC-derived EVs affect angiogenesis [59]. Importantly, the pretreatment with RNase into MSC-EVs abolished the trophic effects [59].

Reis et al. reported that rat bone marrow MSC-derived EV treatment improves renal function in gentamicin-induced AKI by increasing tubular cell proliferation, suppressing cell apoptosis and necrosis, and inhibiting renal inflammation [60]. With MSC-EV treatment, proinflammatory cytokines decrease while anti-inflammatory cytokines increase. Likewise, Wang et al. reported that MSC-EV treatment ameliorates I/R-induced AKI by reducing inflammatory cytokines such as IL-1*β* and TNF-*α* [61], indicating that the regulation of inflammation is one of the major mechanisms for renal protection by MSC-EVs.

Ju et al. reported that human umbilical cord MSC-derived EV treatment protects from I/R-induced AKI by increasing ERK1/2 expression and HGF, which promotes tubular cell dedifferentiation and growth [62]. RNase treatment abolishes these trophic effects, suggesting that RNAs in MSC-EV are essential factors for renoprotection. Gu et al. reported that human umbilical cord MSC-derived EV treatment attenuated AKI-induced renal injury by preserving mitochondrial morphology that was paralleled with reduced apoptosis. They also revealed that miR-30 antagomirs dramatically reduced these protective effects [63]. Furthermore, Collino et al. reported that miRNA deletion in MSCs and EVs reduces regenerative effects in glycerol-induced AKI [64]. These reports imply the critical role of miRNAs in MSC-EVs on promoting renal protection and regeneration after AKI.

Wang et al. reported improved renal function with the treatment of umbilical cord MSC-derived EVs in cisplatin-induced AKI [65]. They revealed that MSC-EV treatment prevented renal injury through the activation of autophagy, and the effect was abolished with the autophagy inhibitor, 3-methyladenine. In the same line, Jia et al. recently reported that umbilical cord MSC-derived EV treatment prevents cisplatin-induced renal injury through the activation of autophagy via trophic factor 14-3-3*ζ* which interacts with ATG-16L [66], indicating proteins in MSC-EVs also contribute to renal protection. Taken together, the activation of autophagy is one of the important mechanisms by MSC-EV treatment.

In addition to the bone marrow-derived and umbilical cord-derived MSC analyses, MSCs from different origins were also examined. Choi et al. applied EVs from kidney-derived MSCs in I/R-induced AKI mice, revealing the trophic effects through the increased cell proliferation as well as the inhibition of cell apoptosis [67]. Lin et al. applied adipose-derived MSCs in I/R-induced AKI and revealed that the combination of adipose-derived MSC-EVs and MSC treatment protects from renal injury after AKI through the inhibition of cell apoptosis, oxidative stress, and renal fibrosis [68]. These reports suggest MSC-EVs from different origins also provide renal protection and regeneration.

**Figure 1:**
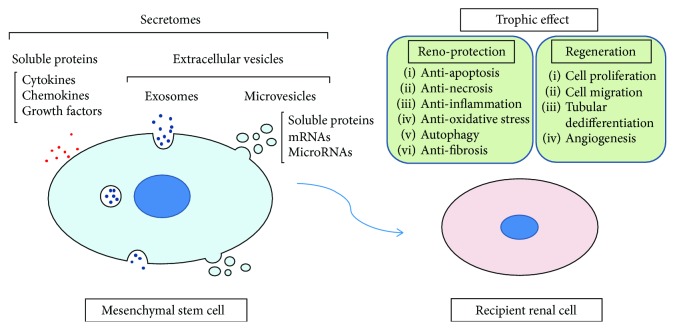
Schema of trophic mechanisms via secretomes from mesenchymal stem cells.

Taken together, renoprotective effects by MSC-derived secretomes might be mainly mediated by EV-containing mRNA, miRNA, and proteins through a variety of mechanisms. Importantly, these trophic mechanisms from MSCs can be divided into two types, renal protection and regeneration after injury. Renal protection is mainly mediated through the suppression of cell apoptosis, cell necrosis, renal fibrosis, renal inflammation, and oxidative stress as well as the promotion of autophagy. Regeneration is mediated through the increase in cell proliferation, migration, tubular cell dedifferentiation, and angiogenesis. Secretomes from MSCs and the trophic mechanisms against renal injury are summarized (Figure 1). Further analyses are required to elucidate the detailed mechanisms by which MSC-EVs protect the kidney from AKI.

## 8. Heterogeneity of Secretomes from MSCs by Different Origins

While a number of studies have identified the secretomes from MSCs for renal protection, the origin of MSCs might affect the types and quantities of trophic secretomes. As described above, Nargesi et al. reported the heterogeneity of mRNAs in MSC-EVs from bone marrow-derived MSCs and adipose-derived MSCs. They indicate that less than 3% of mRNA expression is overlapped between these 2 types of MSCs [28]. Lindsay et al. reported that human MSCs from the olfactory mucosa but not from the bone marrow enhance central nervous system myelination, implying the difference of secretomes from MSCs from different origins [69]. They also reported the different quantities of miRNAs between human olfactory mucosa-derived MSCs and bone marrow-derived MSCs [70]. Using the analysis of miRNA analysis, they showed that 64% of miRNAs isolated from both MSCs were equivalently expressed while 26 miRNAs showed different amounts of expressions, especially in the expression of miR-140-5p and miR-146a-5p, which regulate inflammatory cytokines, such as CXCL12, IL-6, IL-8, and CCL2. These data strongly suggest the heterogeneity in the point of secretomes from different sources of MSCs, which may affect the regenerative effect in MSC-based therapy. Because miRNAs are known to act as regulatory signals for maintaining stemness, self-renewal, and differentiation in adult stem cells, the characterization of miRNAs from MSCs from different tissues may give us insight into what makes the different biological activities [71]. Indeed, it is known that the signaling pathways involved in cell fate specification, including Wnt, BMP, Notch, and TGF-*β* pathways, are associated with MSC differentiations [72–74]. miRNAs are reported to control the balance between self-renewal and differentiation [75]. Lazzarini et al. reported that MSCs isolated from skin and amniotic fluid shared the expression of core miRNAs related to stemness and authorize the definition of MSCs while there were significant differences in the expression of miRNAs associated with adipogenesis, indicating the existence of tissue specificity [76].

Although MSCs from different origins have similarities, characteristic differences have been reported. For example, placental MSCs have superior migratory capacity but less adipogenic potential [77, 78]. Umbilical cord-derived MSCs do not express tumor-associated fibroblast phenotypes [79] and thus have no opportunity to grow tumors. Adipose-derived MSCs possess a higher potential for angiogenesis and vasculogenesis as well as adipogenesis [80]. Tsai et al. applied a microarray comparison between amniotic fluid-, amniotic membrane-, and cord blood-derived MSCs, revealing specific biological functions for MSCs from different origins [81]. In addition, Pelekanos et al. compared MSC-like cells from the heart, bone marrow, and kidney [82] and reported that these 3 types of MSCs share morphological and molecular characteristics as well as multipotency while there are differences in the expression of organ-specific genes to maintain the “memory of tissue origin” reflective of their unique ontogeny and functional roles. They revealed the increased expressions of *Mylk*, *Myom*, *Desmin*, and *Serpinb2* in kidney-derived MSCs, suggesting a strong relationship with the perivascular and mesangial cells, indicating kidney-specific gene expressions. Taken together, there might be distinct functional roles of MSCs isolated from different tissues.

What makes the differences in MSCs from different origins? One of the essential differences between MSCs from different origins might be their cellular niche. The fate of stem cells might be regulated by their microenvironment [83], which might provide the tissue specificity. The transmembrane cell adhesion proteins, cadherins, act in cell-cell adhesion, differentiation, and polarity in MSCs and interact with Wnt, which are involved in the MSC niche [83]. The location of MSCs is associated with how they interact with these molecules, and it would affect their functions. Because MSCs interact with other niche cells both locally and systemically [84], the difference of MSC niche might make the difference of secretomes from MSCs. In addition, the bone marrow milieu is hypoxic in nature. The oxygenic difference also makes the characteristic change of MSCs from the bone marrow and others.

Despite the heterogeneity of secretomes from MSCs isolated from different tissues, there are few papers focusing on the different secretomes of MSCs from different origins. Further analysis and comparison are necessary for elucidating the heterogeneity of secretomes from different MSCs.

## 9. Inducible Secretomes from MSCs by Different Stimuli

In addition to the difference of MSC origin, increasing evidences have proposed the changes of secretomes by different stimuli into MSCs that include aging, hypoxia, inflammatory stimuli, 3-dimensional (3D) culture condition, microparticle stimuli, nanosilicate stimuli, and TGF-*β* stimuli (Table 4). Bustos et al. demonstrated the age-dependent decrease in the expressions of several cytokine/chemokine receptors, which diminish the cell migration and activation of MSCs [85]. They showed the decreased expressions of TNFR, IFNGR, and CCR7 in aged bone marrow-derived MSCs, which might result in the decrease in protective potential. Fafián-Labora et al. explored the difference in the point of miRNA expressions in MSC-EVs, revealing the age-dependent decrease in miR-146a, miR-155, and miR-132 expression and increase in miR-335 expression [86].

Hypoxic condition affects a number of gene transcriptions via stabilization of HIF-1*α*, including angiogenic factors such as VEGF [87]. Song et al. performed the proteomic analysis of MSC secretomes under hypoxia [88], revealing the significant difference in the expressions of 66 out of 231 proteins between the normoxic and hypoxic conditioned media. Especially, they indicated the dramatic increase in 2 tropomyosin isoform expressions in hypoxic condition in a HIF-1*α*-dependent manner. They hypothesize according to the GeneMANIA network analysis that tropomyosin might activate NOS3, which is known to protect against ischemic injury [89]. In addition, Ceradini et al. reported that HIF-1*α*-induced SDF-1 expression in MSCs mediates the recruitments of MSCs to the sites of injured tissue [90]. Furthermore, Crisostomo et al. reported the increased expressions of FGF-2, HGF, and IGF-1 from bone marrow-derived MSCs as well as VEGF in an NF*κ*B-dependent manner under hypoxia [91]. In addition to the secretomes from MSCs, hypoxic condition keeps MSCs in an undifferentiated phenotype for self-renewal. Hypoxic condition increases the expression of stem cell markers, such as Oct-4 and Rex-1 from MSCs, indicating the increased stemness [92]. Hypoxic condition also affects the secretomes related to inflammation. Munn and Mellor reported that the hypoxic condition increases the expression of IDO from MSCs, which regulates the immune system through limiting T cell function and engaging mechanisms of immune tolerance [93]. In summary, hypoxic condition increases the secretomes from MSCs that are associated with angiogenesis and inflammation.

Inflammatory stimuli also affect the secretomes from MSCs. For example, IFN-*γ* treatment into MSCs increases IDO expression [94], which results in the inhibition of MSC cell proliferation as well as the inhibition of the differentiation potential of MSCs into neuron and adipocyte. IFN-*γ* also upregulates the expression of PGE-2 [95]. Inflammatory stimuli by IFN-*γ*, TNF-*α*, and TLR signals increase the expressions of Gal-9 from MSCs [96]. TNF-*α* also increases the expression of BMP-2, which leads to the increase in cell proliferation, migration, and osteogenic differentiation [97]. Like hypoxic condition, TNF-*α* and lipopolysaccharide (LPS) treatments increase the expressions of VEGF, FGF-2, HGF, and IGF-1 from MSCs [91]. Xing et al. reported that inflammatory stimuli increase chemokine secretion to promote the MSC recruiting capacity [98]. They showed the significant increase in cytokine and chemokine expressions including CXCL-16, GRO, ENA-78, MIP-1-delta, osteoprotegerin, MCP-1, MCP-2, MCP-3, IL-6, GCP-2, and IL-2R*α* in proinflammatory treated (IL-1*β*, IL-6, IL-20, and TNF-*α*) culture supernatant compared to nontreated culture supernatant during MSC culture. They also indicated the increased migration of MSCs in the proinflammatory factor-treated culture medium, suggesting that increased soluble factors affect the recruiting ability of MSCs. Overall, inflammatory stimuli increase the regenerative process through the enhanced release of secretomes from MSCs.

Wang et al. applied proteomic profiling to explore the expression pattern of soluble proteins from MSCs with the stimuli of TGF-*β*1 [99], revealing the secreting differences in around 30 proteins with TGF-*β*1 treatment, including cytoskeletal factors (e.g., T-platin, gelsolin), matrix synthesis factors (e.g., collagen-binding protein 2), membrane proteins (e.g., annexin A6, annexin A2), and metabolic enzymes (e.g., thioredoxin reductase, transaldolase, and malate dehydrogenase). They also demonstrated that the decreased expression of gelsolin with TGF-*β*1 treatment enhances the assembly of *α*-actin and actin filaments, which might lead to MSC differentiation. Hence, we need to point out that these different stimuli not only affect the secretomes from MSCs but also affect MSC differentiation.

3D culture systems, such as spheroid culture also, affect the secretomes from MSCs. Bartosh et al. reported the increased expressions of TSG-6 as well as STC1 and three anticancer proteins, TRIL, IL-24 and CD82, from MSCs when they are grown in 3D spheroids [100]. They also revealed that the assembly of MSC into spheres triggers caspase-dependent IL-1 signaling and the secretion of modulators of inflammation [101]. In addition, Frith et al. indicated the increased expression of IL-24 from MSCs cultured in dynamic 3D culture condition compared to monolayer culture condition [102]. These data indicate the importance of the niche and/or environment of culture condition in the point of secretomes from MSCs.

More recently, Carrow et al. reported that human MSCs show widespread change of secretomes when they are stimulated by two-dimensional nanosilicates [103]. They revealed the change of more than 4000 gene expressions by nanosilicate exposure using high-throughput sequencing (RNA-seq). Nanosilicate attaches to the cell membrane, internalizes the cells, and activates stress-response pathways including MAPK, which also affects MSC differentiation toward bone and chondrogenic tissue. This information about the change of secretomes with different stimuli is important for the stable and high-quality supply of secretomes for the cell-free therapy by MSC-EVs against tissue injuries.

## 10. Conclusion

In this review article, we summarize the current evidence about the secretomes from MSCs and the therapeutic mechanisms against AKI. There are several advantages with the use of MSC-EVs. MSC-EVs are the cell-free sources for tissue regeneration which might be safer rather than using MSC cells themselves. In addition, EVs are the sources for cell-cell communications, which might be easily transferred into recipient cells with lower concentration of the factors. Because the renoprotective effect of MSCs against AKI is mainly mediated by MSC-EVs, it may open a new strategy to treat against AKI. There might be the heterogeneity of secretomes of MSCs that depend on the cell origin of MSCs and different stimuli, including hypoxia, inflammatory signals, and aging. Therefore, we need to explore the change of secretomes and further specify the trophic factors, so that we can identify the best sources and factors for the therapy with MSC-EVs. It can also help to get stable and high-quality secretomes from MSCs. In summary, MSC-EV treatment is the promising therapeutic option for the renal protection and regeneration after AKI. Although further analysis and experiments are necessary to develop this therapy, it would open a new strategy to treat against AKI.

## Figures and Tables

**Table 1:** Soluble factors from MSCs.

*Cytokines*	*Chemokines*
IL-1*α*	CCL1
IL-1*β*	CCL2
IL-2	CCL5
IL-3	CCL8
IL-6	CCL11
IL-7	CCL15
IL-10	CCL16
IL-11	CCL18
IL-12	CCL22
IL-13	CCL23
IL-16	CCL24
IFN-*γ*	CCL26
TNF-*α*	CXCL1
LIF	CXCL2
TGF-*β*	CXCL3
MIF	CXCL5
OSM	CXCL6
G-CSF	CXCL8
M-CSF	CXCL11
GM-CSF	CXCL12
FLT3LG	CXCL13
SCF	CX3CL1
Thrombopoietin	XCL1
TSG-6	
	*Other factors*
*Angiogenic factors*	*CXCR3*
Angiogenin	PGE2
Angiopoietin	PAI-1
VEGF	MMP1
	MMP3
*Growth factors*	MMP9
HGF	MMP10
EGF	MMP13
IGF-1	TIMP-1
FGF-2	TIMP-2
FGF-4	TIMP-3
FGF-7	TIMP-4
FGF-9	Leptin
BMP-7	IGFBP-1
BDGF	IGFBP-2
GDNF	IGFBP-3
NGF	IGFBP-4
PIGF	Adiponectin
PDGF	Adrenomedullin
	Osteoprotegerin

IL: interleukin; IFN: interferon; TNF: tumor necrosis factor; LIF: leukemia inhibitory factor; TGF: transforming growth factor; MIF: macrophage migration inhibitory factor; OSM: oncostatin M; G-CSF: granulocyte colony-stimulating factor; M-CSF: macrophage colony-stimulating factor; GM-CSF: granulocyte macrophage colony-stimulating factor; FLT3LG: Fms-related tyrosine kinase 3 ligand; SCF: stem cell factor; TSG-6: TNF-stimulated gene 6; VEGF: vascular endothelial growth factors; HGF: hepatocyte growth factor; EGF: epidermal growth factor; IGF: insulin-like growth factor; FGF: fibroblast growth factor; BMP: bone morphogenetic protein; BDNF: brain-derived neurotrophic factor; GDNF: glial cell-derived neurotrophic factor; NGF: nerve growth factor; PIGF: placenta growth factor; PDGF: platelet-derived growth factor; CCL: C-C motif chemokine ligand; CXCL: C-X-C motif chemokine ligand; CX3CL: C-X3-C motif chemokine ligand; XCL: X-C motif chemokine ligand; CXCR: C-X-C motif chemokine receptor; PGE2; prostaglandin E2; PAI: plasminogen activator inhibitor; MMP: matric metalloproteinase; TIMP: tissue inhibitor of metalloproteinase; IGFBP: insulin-like growth factor-binding protein.

**Table 2:** miRNAs in MSC-derived EVs.

Reference	[30]	[35]	[31]	[32]	[33]	[27]	[34]	[36]	[37]	[38]
miRNA										
miR-1246	○									
miR-23a	○									
miR-451a	○			○					○	
miR-125b	○									
miR-199a	○									
let-7a	○							○	○	
miR-4454/7975	○									
miR-21	○						○		○	
let-7b	○				○					
miR-100	○									
miR-29a	○	○	○							
miR-144	○									
miR-29b	○	○								
miR-22	○						○			○
miR-630	○			○						
miR-221	○									
let-7i	○									
miR-424	○									
miR-191	○						○			
miR-25	○									
miR-130a	○									
miR-376a	○									
miR-27b	○									
miR-30		○								
miR-210		○								
miR-24			○							
miR-1202				○						
miR-638				○						
miR-148a						○		○		
miR-532						○				
miR-378						○				
let-7f						○				
miR-486							○			
miR-10a							○			
miR-10b							○			
miR-222							○			
miR-143							○		○	
miR-199b								○		
miR-218								○		
miR-135b								○		
miR-203								○		
miR-219								○		
miR-299								○		
miR-302b								○		
miR-145									○	
miR-338									○	
miR-1260									○	
miR-1908									○	

**Table 3:** Summary of studies using MSC-derived EV treatment against AKI.

Cause of AKI	Species	MSC origin	EV dose	Route	Outcome	Therapeutic mechanism	Molecules	Reference
Glycerol	Mouse	Human bone marrow	15 *μ*g	IV	(i) Improved renal function	(i) Cell proliferation (ii) Antiapoptosis	RNA	Bruno et al. 2009 [26]
Glycerol	Mouse	Human bone marrow	165 × 10^6^ EV	IV	(i) Improved renal function	(i) Cell proliferation (ii) Antinecrosis	—	Bruno et al. 2017 [52]
Glycerol	Rat	Human bone marrow	2.2 × 10^8^ EV	IV	(i) Improved renal function	(i) Anti-inflammation	miRNA	Collino et al. 2015 [64]
Cisplatin	Mouse	Human bone marrow	100 *μ*g	IV	(i) Improved renal function	(i) Antiapoptosis	—	Bruno et al. 2012 [53]
Cisplatin	Rat	Human umbilical code	200 *μ*g	Renal capsule	(i) Improved renal function	(i) Antiapoptosis (ii) Anti-inflammation (iii) Activation of autophagy	—	Wang et al. 2017 [65]
Cisplatin	Rat	Human umbilical cord	200 *μ*g	Renal capsule	(i) Improved renal function	(i) Activation of autophagy	Protein (14-3-3*ζ*)	Jia et al. 2018 [66]
Cisplatin	Rat	Human umbilical cord	200 *μ*g	Renal capsule	(i) Decreased tubular injury (ii) Improved renal function	(i) Antiapoptosis (ii) Antinecrosis (iii) Antioxidative stress	—	Zhou et al. 2013 [55]
Gentamicin	Rat	Rat bone marrow	—	IV	(i) Improved renal function	(i) Cell proliferation (ii) Antiapoptosis (iii) Antinecrosis (iv) Anti-inflammation	RNA	Reis et al. 2012 [60]
I/R	Rat	Rat bone marrow	100 *μ*g	IV	(i) Improved renal function	(i) Antiapoptosis (ii) Anti-inflammation	—	Wang et al. 2014 [61]
I/R	Rat	Human bone marrow	30 *μ*g	IV	(i) Decreased tubular injury (ii) Improved renal function	(i) Antiapoptosis (ii) Cell proliferation	RNA	Gatti et al. 2011 [54]
I/R	Rat	Human umbilical cord	100 *μ*g	IV	(i) Improved renal function	(i) Cell proliferation (ii) Antiapoptosis (iii) Antifibrosis (iv) Antioxidative stress	—	Zhang et al. 2014 [57]
I/R	Rat	Human umbilical cord	100 *μ*g	IV	(i) Improved renal function	(i) Antiapoptosis (ii) Antinecrosis (iii) Antioxidative stress	—	Zhang et al. 2016 [56]
I/R	Rat	Human umbilical cord	100 *μ*g	IV	(i) Improved renal function	(i) Anti-inflammation (ii) Cell proliferation (iii) Antifibrosis	miRNA	Zou et al. 2014 [58]
I/R	Rat	Human umbilical cord	100 *μ*g	IV	(i) Decreased tubular injury (ii) Improved renal function	(i) Antiapoptosis (ii) Cell proliferation (iii) Antifibrosis (iv) Angiogenesis	RNA	Zou et al. 2016 [59]
I/R	Rat	Human umbilical cord	30 *μ*g	IV	(i) Improved renal function	(i) Proangiogenic factors (ii) Antifibrosis (iii) Antiapoptosis (iv) Tubular dedifferentiation	RNA	Ju et al. 2015 [62]
I/R	Rat	Human umbilical cord	100 *μ*g	IV	(i) Improved renal function	(i) Antiapoptosis (ii) Preserved mitochondrial morphology	miRNA (miR-30)	Gu et al. 2017 [63]
I/R	Mouse	Mouse kidney resident	2 × 10^7^ MSCs	IV	(i) Improved renal function	(i) Antiapoptosis (ii) Cell proliferation	—	Choi et al. 2014 [67]
I/R	Rat	Rat adipose tissue	100 *μ*g	IV	(i) Improved renal function	(i) Antiapoptosis (ii) Antioxidative stress (iii) Antifibrosis (iv) Angiogenesis	N/A	Lin et al. 2016 [68]

AKI: acute kidney injury; MSC: mesenchymal stem cell; EV: extracellular vesicles; I/R: ischemia/reperfusion; IV: intravenous.

**Table 4:** Summary of studies about secretome changes from MSCs in different stimuli.

Stimuli	Increased secretomes	Reference
Hypoxia	Tropomyosin, VEGF, SDF-1, FGF-2, HGF, IGF-1, Oct-4, Rex-1, and IDO	[88, 90–93]
IFN-*γ*	IDO, PGE-2, and Gal-9	[93–96]
TNF-*α*	Gal-9, BMP-2, VEGF, SDF-1, FGF-2, HGF, and IGF-1	[91, 96, 97]
TLR signal	Gal-9	[96]
Inflammatory stimuli (IL-1*β*, IL-6, IL-20, and TNF-*α*)	CXCL-16, GRO, ENA-78, MIP-1-delta, osteoprotegerin, MCP-1, MCP-2, MCP-3, IL-6, GCP-2, and IL-2RA	[98]
LPS	VEGF, SDF-1, FGF-2, HGF, and IGF-1	[91]
3D culture	TSG-6, STC1, TRIL, IL-24, and CD82	[100–102]
Nanosilicate	More than 4000 gene expression change	[103]
Aging	Increase: miR-335	[85, 86]
	Decrease: TNFR, IFNGR, CCR7, miR-146a, miR-155, and miR-132	
TGF-*β*	Cytoskeletal factors (e.g. T-platin, gelsolin)	[99]
	Matrix synthesis factors (e.g., collagen-binding protein 2)	
	Membrane proteins (e.g., annexin A6, annexin A2)	
	Metabolic enzymes (e.g., thioredoxin reductase, transaldolase, and malate dehydrogenase)	

LPS: lipopolysaccharide; 3D: 3-dimensional.
